# *In Vitro* Maturation, Fertilization and Embryo Culture of Oocytes Obtained from Vitrified Auto-Transplanted Mouse Ovary

**Published:** 2013-03-03

**Authors:** Arash Behbahanian, Hossein Eimani, Bahman Zeinali, Mojtaba Rezazadeh Valojerdi, Poopak Eftekhari Yazdi, Abdolhossein Shahverdi, Hamid Gourabi, Afsaneh Golkar-Narenji

**Affiliations:** 1 Department of Embryology at Reproductive Biomedicine Research Center, Royan Institute for Reproductive Biomedicine, ACECR, Tehran, Iran; 2 Department of Biology, Faculty of Basic Sciences, Tehran University, Tehran, Iran; 3 Department of Anatomy, Faculty of Medicine, Baqiyatallah (A.S.) University of Medical Sciences, Tehran, Iran; 4 Department of Genetics at Reproductive Biomedicine Research Center, Royan Institute for Reproductive Biomedicine, ACECR, Tehran, Iran

**Keywords:** *in vitro* Maturation, Ovary, Vitrification, Oocytes

## Abstract

**Background:**

The purpose of this study was to investigate the *in vitro* survival and developmental potential of oocytes obtained from vitrified mouse ovaries transplanted to a heterotopic site.

**Materials and Methods:**

In this experimental study, two-week-old mice were unilaterally ovariectomized after anesthesia. The ovaries were vitrified by cryotop. After two weeks, the ovaries were thawed and autotransplanted to the gluteus muscle tissue. Three weeks later the mice were killed, after which we removed and dissected the transplanted and opposite right ovaries. Cumulus oocyte complexes (COCs) and denuded oocytes were evaluated for *in vitro* maturation (IVM), in vitro fertilization (IVF) and *in vitro* development (IVD). The control group consisted of sevenweek- old age-matched mice ovaries.

**Results:**

All vitrified-transplanted (Vit-trans) ovaries contained some oocytes that survived. Following IVM, IVF and IVD, there were 41.7% out of 12 cultured zygotes that reached the 8-cell stage.

**Conclusion:**

Our experiment supports the progressive role of long-term graft survival after wholeovarian cryopreservation by vitrification and subsequent heterotopic transplantation. It is possible to recover viable follicles and oocytes that have the ability to develop *in vitro*.

## Introduction

Several potential options are available to preserve fertility in patients that face premature ovarian failure, including immature and mature oocyte, and embryo cryopreservation. Each direction has its own benefits and limitations. In cases where chemotherapy cannot be postponed, cryopreservation of ovarian tissue is an option (1,2). A large number of follicles at different developmental stages are present in mammalian ovaries and the ovarian cortex of young women can be considered a potential storage source for oocytes that could be used for clinical, agricultural, and zoological purposes (3). Thus, as the oocytes of primordial follicles are less liable to cytogenetic errors, they are favorable for cryopreservation (4-6). Studies on murine models have shown development of primordial to preantral follicles in vivo, followed by *in vitro* growth (IVG), *in vitro* maturation (IVM), fertilization and further development (7,8). Cryopreserved primordial follicles in their natural environment can be used for fertility preservation in different ways. Orthotopic transplantation of fresh or cryopreserved ovarian tissues have resulted in recovery of folliculogenesis, steroidogenesis, ovulation, and fertilization followed by the production of embryos or the delivery of live young (9,10). There are three ways to utilize frozen-thawed ovarian tissue: heterotopic autografting, orthotopic autografting and xenografting. Ovarian tissue could be transplanted to the site of the original tissue (orthotopic) or it can be at a heterotopic site followed by *in vitro* fertilization (IVF). Experiences on autotransplantation of cryopreserved ovarian tissue have shown that this method can restore endocrine function and fertility in animals (10) and humans (11).

Autotransplantation of cryopreserved ovarian tissue offers the possibility of restoring ovarian function in women and children after highly gonadotoxic cancer treatment. Theoretically, orthotopic autotransplantation can restore normal reproductive function, which leads to natural conception. A live birth in a primate following a fresh ovarian tissue transplant and the first live birth in a human after orthotopic transplantation of cryopreserved ovarian tissue have been reported in 2004 (12). After completion of chemotherapy treatment, the ovarian tissue is considered for reimplant. Tissue ischemia after grafting, however, can cause a significant loss of follicles (13) and consequently shorten the life span of ovarian grafts. The transplantation site is one of the most important factors (14). The reimplantation site can be orthotopic (pelvic cavity) or heterotopic (forearm) (15), beneath the abdominal skin (16), and pelvic side wall (17). We have shown previously that the back muscle is an available transplantation site for follicle survival and development in mice (18). As with other groups, this location has been proven to be a promising site for ovarian tissue transplantation (19). However, further investigations and experiments are necessary for this location to become a suitable site for transplantation. The aim of this experiment is to investigate the developmental potential cumulus oocyte complexes (COCs) derived from cryopreserved-transplanted ovaries.

## Materials and Methods

In this experimental study, all chemicals were purchased from Sigma company (Germany), except those mentioned below.

### Animals

Animal experiments were performed according to the Declaration Helsinki and the Guiding Principles in the Care and Use of Animals (DHEW publication, NIH, 80-23).

For the experiments, we used female NMRI mice that were housed, bred and kept at a temperature of 20-25°C and 50% humidity under light-controlled conditions (12 hour light/12 hour dark) and provided with sterile food and water in the Central Animal House of Royan Institute according to national standards.

### Experimental groups

We euthanized two-week-old mice, after which their left ovaries were removed and immediately placed in α-MEM medium at room temperature. The fat tissue surrounded the ovaries were removed under a loop (5 minutes). Then, we immediately collected the ovaries to perform the cryopreservation procedure. After two weeks of cryopreservation, whole ovaries were thawed and autotransplanted to the same mouse from which the ovary had been removed. Three weeks after transplantation of thawed ovaries, the seven-week-old mice were killed and the grafted ovaries on the gluteus muscle tissue were collected. These ovaries were considered the experimental group (Vit-trans group). In addition to the collection of grafted ovaries, we also collected the right ovaries from the same mice, which were considered to be the opposite ovaries (Opp) group. For the control group, both ovaries of age matched (seven-week-old) female mice were collected (7 week-fresh group).

### Ovary vitrification and thawing

**Fig 1:**
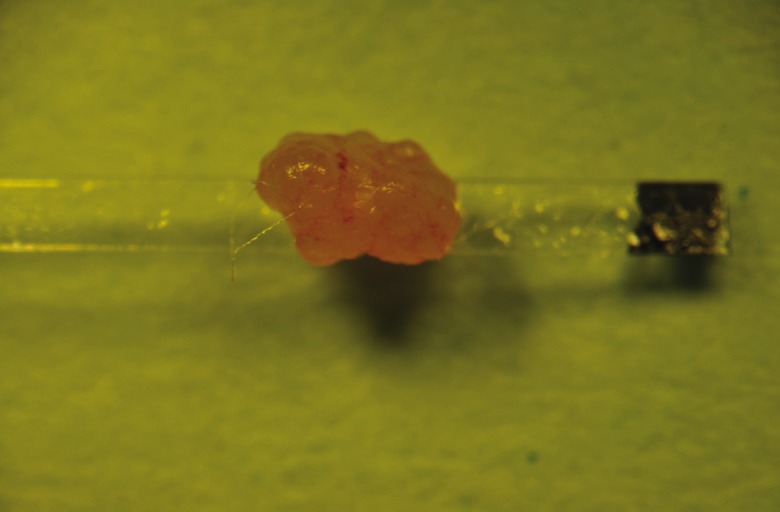
Whole mouse ovary on the Cryotop polyester sheet.

Following ovariectomy, the ovaries were immediately vitrified. During vitrification, ovaries were immersed in an equilibration solution composed of 7.5% DMSO and 7.5% ethylene glycol (EG) in HEPES-buffer TCM 199 (Gibco, USA; pH=7.4) supplemented with 20% human serum albumin (HSA) (Octapharma, Switzerland) for 15 minutes at room temperature. Ovaries were subsequently transferred into the vitrification solution that consisted of 15% EG, 15% DMSO, and 0.5 M sucrose in HEPES-buffer TCM 199 (pH=7.4) supplemented with 20% HSA for 30 minutes. Each whole ovary was placed on the Cryotop (Kitazato Co., Ltd., Fujinomiya, Japan) polyester sheet with a minimum volume of the vitrification solution (Fig 1). The sheets were plunged immediately into liquid nitrogen and capped. After placing the cryotops in their cassettes, we transferred them to a nitrogen tank where they remained for two weeks.

For thawing, after pulling up the cryotops from liquid nitrogen, the sheets were placed directly in a thawing solution composed of 1 M sucrose in HEPES-buffer TCM199 (pH=7.4) supplemented with 20% HSA for 10 minutes at room temperature. The ovaries were detached from the sheet and transferred to α-MEM medium (Gibco, USA) supplemented with 10% fetal bovine serum (FBS) and an antibiotic solution composed of penicillin G (100 IU) and streptomycin (100 IU) after which ovaries were incubated for 30 minutes.

### Transplantation

Thawed ovaries were immediately transplanted back into the same female mouse which had her left ovary removed two weeks prior. At this time, we anesthetized four-week-old mice with intraperitoneal injections of 10% ketamine (100 mg/ kg; Alfasan, Woerden, Holland) and 2% xylazine (10 mg/kg; Alfasan, Woerden, Holland). The concentrations of 100 mg/ml Ketamine and 20 mg/ml Xylazine were diluted in 8.5 ml physiological serum; 0.1 g body weight was injected.

After a full incision through the dermal tissue and the placement of an incision along the gluteus superficialis muscle fibers, the ovary was inserted within the muscle incision. The muscle fiber was sutured with 5-0 non-absorbable vicryl surgical thread (Ethicon, Belgium) in order to allow detection of the transplantation site. The skin was sutured with 6-0 absorbable threads (prolene; Ethicon, Belgium). Following the grafting procedure and recovery, animals were kept in the animal room.

### Gonadotropin treatment

About 72 hours prior to ovary removal, we injected 7.5 IU/ml PMSG (Folligon, Intervet) in mice from both the experimental and control groups; 48 hours later, both groups received 7.5 IU/ml hCG (Pregnyl, Organon) and after 14 hours we collected the ovaries.

### Graft recovery

At three weeks following transplantation, mice were killed by cervical dislocation. Grafted and Opp were removed from the experimental groups and fresh ovaries were removed from intact mice for oocyte isolation.

### Mechanical dissection and oocyte isolation

Each ovary was mechanically dissected by a 26- gauge needle in α-MEM droplets supplemented with 10% v/v FBS and antibiotic solution (penestrep). Released oocytes and COCs from the ovary were selected for IVM or IVF by the following criteria. Those oocytes and COCs that lacked polar bodies were chosen and divided into two groups: germinal vesicle (GV) and germinal vesicle breakdown (GVBD). Both groups were transferred to IVM culture medium.

The oocytes and COCs that contained polar bodies were selected and transferred to IVF medium. For selection, the oocytes had to be visible and round in shape.

### Oocyte *in vitro* maturation

IVM medium was composed of α-MEM supplemented with 5% FBS, 100 mIU/ml rhFSH (GONAL-f, Serono) and 7.5 IU/ml hCG (Pregnyl, Organon). After a 16 hour incubation period, we observed oocytes and COCs (after pipetting to remove granolusa cells) to determine which had released first the polar bodies. These were assessed as metaphase II (MII) stage oocytes, which were subsequently transferred to IVF medium.

### *In vitro* fertilization (IVF)

We used 7- to 14-week-old adult male mice to obtain sperm. The caudae epididymides were cut in several zones and placed in a 1 ml droplet of T6 medium supplemented with 15 mg/kg BSA. Droplets were covered with mineral oil and incubated for at least 30 minutes at 37°C in a humidified atmosphere with 5% CO_2_ for sperm capacitation. About 10 mature MII oocytes were added to the 100-150 µL droplet of sperm suspension at a concentration of 0.8 × 10^6^ sperm per ml and then incubated for at least 4 hours. The oocytes were pipetted to remove the attached sperm and monitored under a microscope for the presence of a second polar body or two pronuclei (2PN) to confirm fertilization.

### Embryo culture

About 10 fertilized oocytes were cultured in a 20 µl droplet comprised of T6 medium supplemented with 4 mg/ml BSA. Droplets were covered with mineral oil. The developmental stages of the embryos were observed at 24, 48, 72 and 96 hours after fertilization.

### Statistical analysis

All numbers are presented as percentages. Analyses and comparison of significances were performed by the Chi-square test. P<0.05 was considered to be statistically significant.

## Results

All Vit-trans mouse ovaries (n=15) were recovered five weeks following vitrification and transplantation. We collected the Opp ovaries (n=6) and age-matched 7w-fresh mouse ovaries (n=10). There were 66 oocytes, which included 16 COCs and 50 denuded oocytes collected from dissected Vit-trans ovaries. There were 106 (47 COCs, 59 denuded oocytes) oocytes collected from the Opp ovaries and 83 (53 COCs, 30 denuded oocytes) oocytes collected from 7w-fresh ovaries (Table 1).

We recorded the IVM rates of oocytes recovered from transplanted and OPP ovaries for each female mouse (Table 1). We considered each ovary as one replication. After IVM, 43.8% of COCs and 38% of denuded oocytes reached stage MII in the Vit-trans group. The maturation rate of denuded oocytes in Vit-trans group was significantly lower than the 7w-fresh control group (76.7%). There was a significantly lower percentage of MII oocytes following IVM of the Opp group’s denuded oocytes (27.1%) compared to the 7w-fresh group. There was a significant difference in the GVBD rate of Vit-trans (37.5%) and Opp group (10.6%) COCs. Denuded oocytes of the Vit-trans group had a significantly higher GVBD rate of 32.0% compared to Opp (13.6%) and 7w-fresh (10%).

**Table 1:** Oocyte development after *in vitro* maturation


		Maturation stages					
Groups		Total	GV%	GVBD%	MII%	GVBD+MII%	Degenerated

**Vit-trans**	COC	16	12.5	37.5a	43.8	81.3	6.3
	Denuded oocytes	50	18.0	32.0^b, c^	38.0^a^	70.0^a^	12.0
**Opp**	COC	47	12.8	10.6^a^	44.7	55.3^c^	14.9
	Denuded oocytes	59	5.1	13.6^b^	27.1^b^	40.7^a, b^	20.3
**7 week-fresh**	COC	53	20.8	13.2	62.3	75.5^c^	3.8
	Denuded oocytes	30	10.0	10.0^c^	76.7^a, b^	86.7^b^	3.3


MI; Metaphase II, COC; Cumulus oocyte complex, Opp; Opposite ovaries, Vit-trans; Vitrified-transplanted ovaries GV; Germinal vesicle, GVBD; Germinal vesicle breakdown and 7w-fresh; Fresh ovaries removed from 7-week-old normal mice. Percentages with same letters in each column are significantly different (p<0.05).

The number of denuded oocytes that reached GVBD or MII stage in the Vit-trans group (70%) was significantly higher than the Opp group (40.7%). In each group there were a few numbers of degenerated oocytes (Table 1). We observed no significant differences in the percentage of degenerated oocytes among the experimental and fresh groups.

In order to assess the capacity of mature oocytes after vitrification and transplantation, the matured oocytes obtained from Opp and 7w-fresh groups were fertilized and cultured *in vitro*.

There was only a significant difference in fertilization rate and formation of 2PN observed between the Opp (42.9%) and 7w-fresh groups (73.1%). The Vit-trans oocytes had a mediocre rate of IVF (56.5%) between Opp and 7w-fresh groups (Table 2).

**Table 2:** Comparison of in vitro fertilization rate between groups


Groups	Total	2PN(%)*

**Vit-trans**	23	56.5
**Opp**	42	42.9*
**7 week-fresh**	52	73.1*


Vit-trans; Vitrified, transplanted ovaries, 7w-fresh; Fresh ovaries removed from 7-week-old normal mice and 2PN; Two pronuclear. Percentages with same letters in each column are significantly different (p<0.05). *;The percentage of ova with male and female pronucleus.

Cleavage stages were observed until the blastocyst stage, which was approximately 96 hours after fertilization. The results are summarized in table 3.

From a total of 13 zygotes obtained from Vittrans group IVF, 12 zygotes were cultured and 5 embryos (41.7%) reached the 8-cell stage. No additional progress was visualized in this group by 96 hours post-observation. There were no significant differences between Vit-trans, Opp and 7w-fresh groups on the development of embryos to the 8-cell stage after 72 hours of culture (Table 3).

**Table 3:** *In vitro* development of embryos after IVF


		Embryo development							
Experimental Groups	Total	24 hours		48 hours		72 hours		96 hours	
		2-cell (%)	2-cell (%)	4-cell (%)	4-cell (%)	8-cell (%)	8-cell (%)	Morula (%)	Blastocyst (%)

**Vit-trans**	12	91.7	50.0	50.0	0.0	41.7	0.0	0.0^a,b^	0.0^a,b^
**Opp**	15	73.3	13.3	40.0	0.0	40.0	0.0,	40.0^a^	33.3^a^
**7 week-fresh**	38	92.1	23.7	63.2	5.3	60.5	0.0	55.3^a^	47.4^a^


Vit-trans; Vitrified, transplanted ovaries and7 week-fresh; Fresh ovaries removed from 7-week-old normal mice. Percentages with same letters in each column are significantly different (p<0.05).

## Discussion

The major challenge of ovarian tissue transplantation is still ischemia. Attempts have been made to overcome ischemia-related damage that exists at transplantation by treatment with GnRH and antioxidant agents in addition to microvascular anastomosis (20). Through our previous study we have demonstrated that the gluteal muscle is a suitable site for ovarian transplantation (18). The combination of the cryopreservation method using cryotops and transplantation in the gluteal muscle has been evaluated by the histological examination of transplanted ovaries. Histological results indicated that this method increased both the numbers of good quality follicles and angiogenesis (21).

In this research, we evaluated the IVM, IVF and IVD rates of oocytes following cryopreservation of whole ovaries on cryotops and subsequent autotransplantation. During ovarian dissection and oocyte retrieval, there was an elevated rate of denuded oocytes in the Vit-trans and Opp groups compared to the 7wfresh group. The IVM rates of COCs retrieved from Vit-trans and 7w-fresh groups were the same, as was the IVF rate and developmental competence of oocytes that reached the 8-cell stage. The freezing protocol used during this research contained EG. According to Salehnia et al. the vitrification of whole mouse ovaries using EG was useful and had no harmful effects on follicle morphology (22). It has been shown that vitrification of mouse ovarian tissue on a cryotop could highly preserve the viability of ovarian preantral follicles (23). The current research confirmed those previous findings. In a previous experiment, we observed partial restoration of ovarian function following freezing/thawing and heterotopic autotransplantation of whole ovine ovaries. In that research, the IVM of oocytes and subsequent embryo development was successful (24). Another research on the transplantation of whole ovine ovaries resulted in a live birth (25).

During this research the whole ovary was transplanted to the gluteal muscle. We have previously demonstrated that the gluteal muscle is suitable site for ovarian transplantation (18). The same rates of IVM and IVF in both the Vit-trans and 7w-fresh groups have shown that cryopreservation of whole ovaries using a cryotop with subsequent transplantation to the gluteal muscle preserves ovarian function. There were no significant differences in the percentage of arrested oocytes at the GV stage. However the rates of oocytes that reached the GVBD and MII stages in denuded oocytes were lower in the Vit-trans and Opp groups compared to the 7w-fresh group. We did not observe those differences in COCs, which could be attributed to the role of cumulus cells for IVM. It has been shown that the presence of granolusa cells around the oocyte is necessary for uptake of nutrients and essential factors for oocyte development (26), therefore the IVM, IVF and IVD rates of mouse immature oocytes with cumulus cells are higher than denuded immature oocytes (27).

The lower percentage of GVBD and MII oocytes, the higher percentage of degenerated oocytes and lower IVF rates in the Opp group in contrast to other groups might result from the adverse effect of FSH and LH up regulation during two weeks of vitrification and the few days of transplantation followed by hormone therapy in the absence of other ovary. Although the fertilization rate in the Vit-trans group was lower than 7w-fresh group, this difference was not significant. It has been reported that the presence of granolusa cells around the oocytes caused a higher IVF rate (27). Therefore this difference could be the result of a lower COCs/denuded oocyte rate in the Vit-trans group. In the Vit-trans group, embryo development reached the 8-cell stage with no significant difference in the embryo development rate compared to the control group, however this development did not proceed to the morula and blastocyst stages. Possibly, with the use of sequential embryo culture media, the 8-cell embryos might continue IVD to the blastocyst stage. In previous studies, we have shown that vitrification was less deleterious to mouse ovarian tissue in contrast with transplantation ischemia injuries (18, 23). In the present study the most concerning result was the blockage of embryo development at the 8-cell stage, which was possibly the result of suboptimal *in vitro* conditions. According to the previous study which claimed that vitrification caused less injuries in ovarian tissue, it was more probable that this blockage did not result from the vitrification method (23); rather, transplantation injuries and/or in vitro culture problems were more likely explanations.

Despite lower retrieval of COCs from Vittrans ovaries, it seems that for *in vitro* procedures COCs are more competent. Thus it is better to use COCs for *in vitro* embryo production. The main concern with autotransplantation of ovarian tissue in cancer patients is the risk of re-introducing cancer cells and metastasis. Although follicle culture is an alternative, but concerns about the formation and integrity of imprints in oocytes growing and ripening in vitro still remain, which could result in embryonic death or unhealthy offspring (7, 28).

## Conclusion

IVD of generated embryos from Vit-trans ovaries was observed. The combination of cryopreservation (cryotop) and subsequent transplantation on gluteal muscle have been shown to preserve ovarian function. Research is ongoing for ovarian cryopreservation, transplantation and subsequent *in vitro* procedures. Thus, more intensive research on ovarian transplantation and the subsequent *in vitro* procedures is necessary in order to improve the efficiency of this process and its clinical applications.
